# Efficacy of closed cell self expandable metallic stent for peripheral arterial disease in the porcine iliac artery

**DOI:** 10.1038/s41598-023-35878-y

**Published:** 2023-05-26

**Authors:** Dae Sung Ryu, Dong-Sung Won, Ji Won Kim, Yubeen Park, Song Hee Kim, Jeon Min Kang, Chu Hui Zeng, Dohyung Lim, Hyun Choi, Jung-Hoon Park

**Affiliations:** 1 Biomedical Engineering Research Center, Asan Institute for Life Sciences, Asan Medical Center, 88 Olympic-ro 43-gil, Songpa-gu, Seoul, 05505 Republic of Korea; 2 Department of Mechanical Engineering, Sejong University, 209, Neungdong-ro, Gwangjin-gu, Seoul, 05006 Republic of Korea

**Keywords:** Preclinical research, Biomedical engineering

## Abstract

This study aimed to investigate the efficacy of a closed-cell self-expandable metallic stent (SEMS) with or without expanded-polytetrafluoroethylene (e-PTFE)-covering membrane in a porcine iliac artery model. Twelve Yorkshire domestic pigs were divided into a bare closed-cell SEMS (B-SEMS) group (n = 6) and covered closed-cell SEMS (C-SEMS) group (n = 6). Both closed-cell SEMSs were placed in the right or left iliac artery. Thrombogenicity score in the C-SEMS group was significantly higher than that in the B-SEMS group (*p* = 0.004) after 4 weeks. Angiographic findings of mean luminal diameters at 4 weeks follow-up did not differ significantly between B-SEMS and C-SEMS groups. Neointimal hyperplasia thickness as well as degree of inflammatory cell infiltration and collagen deposition in the C-SEMS group was significantly greater than that in the B-SEMS group (*p* < 0.001). Closed-cell SEMSs successfully maintained patency for 4 weeks without stent-related complications in the porcine iliac artery. Although mild thrombus with neointimal hyperplasia was observed in the C-SEMS group, subsequent occlusion, and in-stent stenosis did not occur in any of the pigs until the end of the study. Closed-cell SEMS with or without the e-PTFE covering membrane is effective and safe for the porcine iliac artery.

## Introduction

Peripheral artery disease (PAD) is a widely prevalent vascular disease that affects 202 million people worldwide^1^. PAD dramatically affects quality of life and is associated with significant morbidity and mortality, with an age-adjusted death rate of 17.0 per 100,000 people^2^. Endovascular therapy has evolved into a minimally invasive treatment option to reduce morbidity^3,4^. Balloon-expandable and self-expandable metallic stents (SEMSs) with or without a covered membrane, are widely used to improve the technical success and its patency^4,5^. However, the risks of in-stent restenosis and stent fracture grow with lesion length after placing a bare SMES^6^. A covered SEMS prevents neointimal tissue in-growth and improves stent patency while being less dependent on lesion length. However, covered SEMS are associated with increased risks of stent thrombosis and proximal and distal edge restenosis^7^. To overcome these complications, various antiproliferative drugs, covering materials, stent materials, and stent configurations were investigated^8,9^.

The enormous variety of stent designs with respect to the arrangement of stent cells and the amount of free cell area present between the struts were investigated to prolong stent patency and reduce stent-related complications^8–10^. The two types of cell configuration, closed and open-cell SEMSs, were commonly used for the vascular indications^10^. Closed-cell SEMSs are characterized by interconnected stent struts with small free cell areas, whereas the open-cell SEMSs have relatively large gaps between the struts. Several commercial stents for vascular disease have typically adopted open-cell configuration^11,12^. Theoretically, the closed-cell SEMSs are less flexible and may develop kinks and incomplete expansion. Conversely, open-cell SEMSs conform best to angulated vessels or tortuous anatomy^12^. However, stent-related complications, such as inflammation caused by neointimal proliferation and thrombosis, remain in complex iliac lesions^4,5,13^.

Herein, we developed the closed-cell SEMS using the hand-knitted technique and expanded-polytetrafluoroethylene (e-PTFE) as a covering membrane to evaluate patency, thrombosis, and degree of neointimal hyperplasia formation. The stent materials adopted in this study, nitinol, and e-PTFE, have been used extensively in stent-grafts showing good biocompatibility for the treatment of various vascular diseases^14^. Hence, the purpose of this study was to investigate the efficacy of closed-cell SEMS with or without an e-PTFE membrane in a porcine iliac artery model.

## Methods

### Preparation of closed-cell self-expandable metallic stents

**Figure 1:**
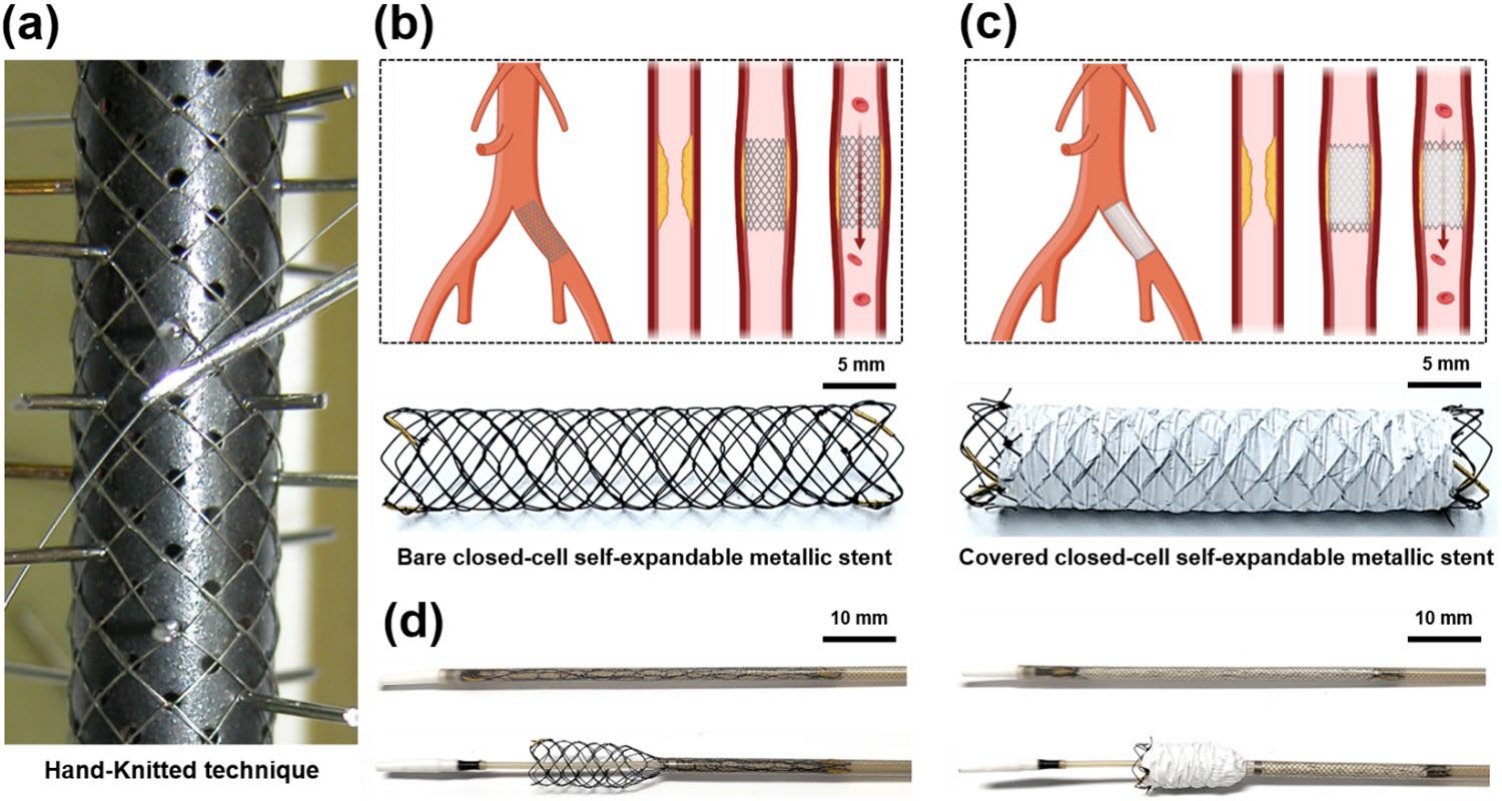
Technical steps of fabricated closed-cell SEMS using a nitinol wire by hand-knitted technique. (**a**) Fabrication of closed-cell SEMS by hand-knitted technique using tubular diamond-cell configuration. (**b**) Closed-cell SEMS without e-PTFE covering membrane. (**c**) Closed-cell SEMS with e-PTFE covering membrane. (**d**) Closed-cell SEMS regardless of the presence of e-PTFE loaded into a 6-Fr delivery system and partially deployed from the braided sheath. Note. SEMS: self-expandable metallic stent, e-PTFE: expanded-polytetrafluoroethylene.

The knitted closed-cell SEMS used in this study was not commercially available. Herein, the closed-cell SEMS (S&G Biotech Co., Ltd., Yongin, Korea) was fabricated using a hand-knitted technique with a single thread of 0.127-mm thick nitinol wire into a tubular diamond-cell configuration (Fig. 1a). The fully expanded SEMS was 6 mm in diameter and 30 mm in length (Fig. 1b,c). The 50 µm thick covering membrane was made of e-PTFE using a weaving fabrication technique, and e-PTFE was sutured to the exterior of the closed-cell SEMS with 5-0 non-absorbable suture (Black-silk, Ailee Co., Ltd., Busan, Korea). The covering membrane partially covered the exterior of the closed-cell SEMS, leaving a 3 mm uncovered portion at both ends (Fig. 1c). Radiopaque markers were attached to both ends and the middle of the stent to facilitate visualization under fluoroscopy. The closed-cell SEMS with or without e-PTFE was loaded into a 6-Fr delivery system using a manual crimping technique. The 6-Fr delivery system had a usable length of 900 mm and comprised an outer braided sheath, a pusher catheter, and a guidewire passing tube with a guiding olive tip. The stents were placed into the arteries using the push–pull deployment technique (Fig. 1d).

### Mechanical properties of closed-cell self-expandable metallic stents

**Figure 2:**
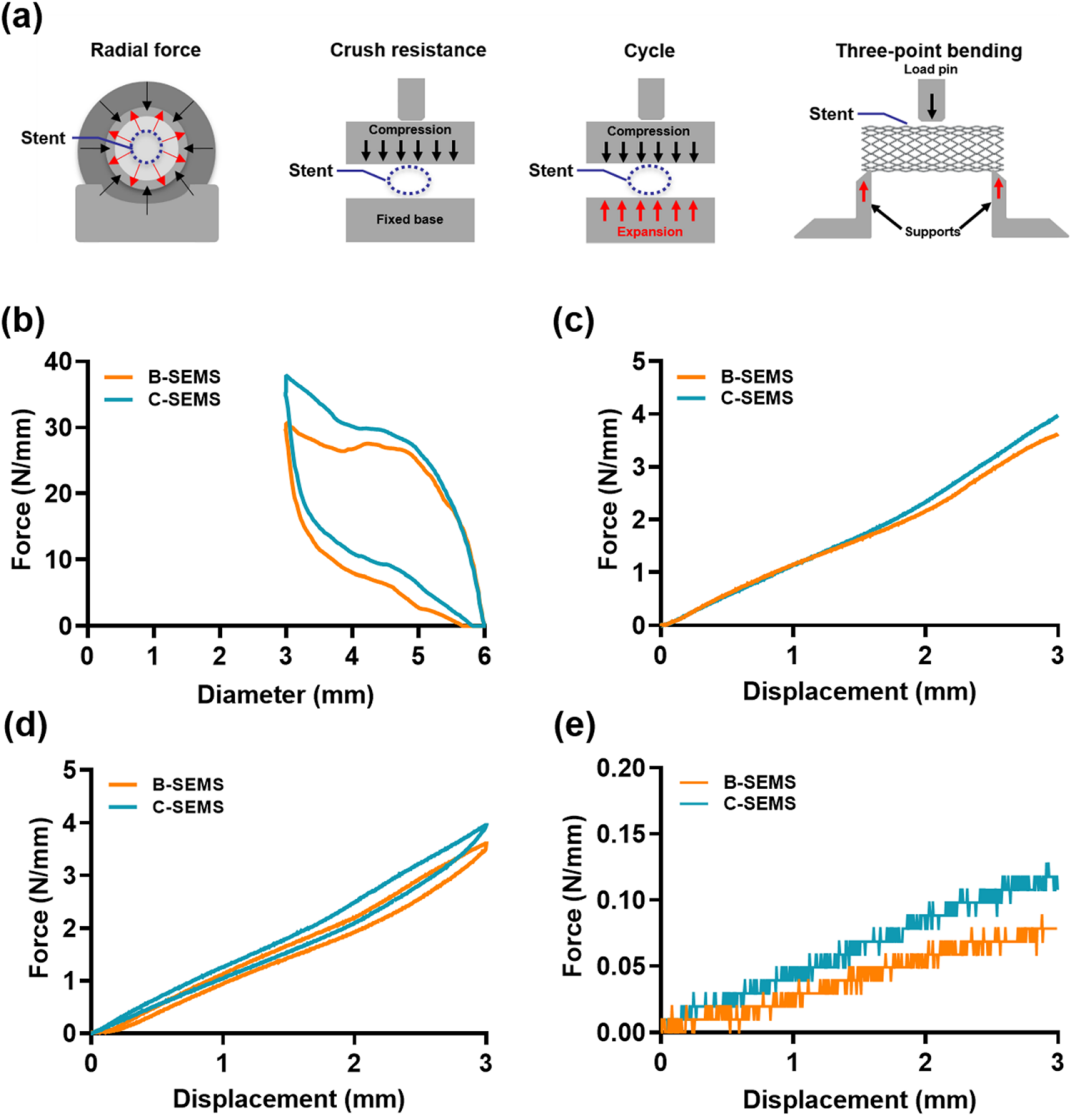
The results of the bench-top tests (**a**) Schematic illustration of the bench-top tests. (**b**) Force-diameter response under radial force, (**c**) force–displacement response under crush resistance, (**d**) cycle, and (**e**) three-point bending characteristics of the closed-cell SEMSs were investigated to confirm the usefulness of the design and hand-knitted technique. Note. SEMS: self-expandable metallic stent.

The closed-cell SEMSs, regardless of the e-PTFE covered membrane, used for mechanical property evaluation were 6 mm in diameter and 30 mm in length. Radial force was assessed using a radial force testing machine (TTR2, Blockwise engineering, Tempe, AZ, USA) from the radial compression and expansion state. The temperature-control die was heated to 37 °C for mimicking in vivo characteristics, and the stent was left in the crimping head for 5 min prior to testing. The tester was programmed to reduce stent diameter from the unconstrained 6 mm to a predetermined 3 mm. Crush resistance, cycle, and three-point bending force tests were conducted using a universal testing machine (UTM; MINOS-005, MTDI, Daejeon, Korea) (Fig. 2a). All measurements for bench-top tests were performed at a speed of 0.5 mm/s. Forces and displacement values were recorded at intervals of 0.05 s on force-diameter and force–displacement graphs. The test was performed using the load-controlled feedback mode with a 30 s linear loading at a peak force of 50 kgf. The loading/unloading cycle test lasted for 60 s^15–18^.

### Animal study design

 A total of 12 iliac arteries of 12 Yorkshire domestic pigs weighing 31.5–36.6 kg (male; mean 33.1 kg; International Animal Experiment Center, Pocheon, Korea) were used in this study and divided into two groups: bare closed-cell SEMS (B-SEMS) group (n = 6), which received closed-cell SEMS without covering membrane placement into the right or left iliac artery; and covered closed-cell SEMS (C-SEMS) group (n = 6), which received closed-cell SEMSs with an e-PTFE-covered membrane into the right or left iliac artery. All animals were housed under the same environmental conditions (temperature of 24 °C ± 2 with a 12 h day-night cycle) and were supplied with water and food ad libitum. All pigs were euthanized with 75 mg/kg potassium chloride (KCl; Dai Han Pharm Co., Seoul, Korea) via marginal ear vein injection 4 weeks after the procedure.

### Closed-cell self-expandable metallic stent placement

The stent placement technique has been previously described in detail^12^. Pigs were placed in the supine position and a sterile surgical technique was used. All pigs were anesthetized intramuscularly with a mixture of 50 mg/kg zolazepam, 50 mg/kg tiletamine (Zoletil 50; Virbac, Carros, France), and 10 mg/kg xylazine (Rompun; Bayer HealthCare, Leverkusen, Germany) before the experiment. An endotracheal tube was then placed and anesthesia was maintained by inhalation of 0.5–2% isoflurane (Ifran®; Hana Pharm. Co., Seoul, Korea) with 1:1 oxygen concentration (510 mL/kg/min). The left femoral artery was cannulated with a 7-Fr arterial sheath (Radifocus; Terumo Co, Tokyo, Japan) under ultrasonographic (iU22; PHILIPS, Amsterdam, Netherlands) and fluoroscopic guidance (FORTE; DK Medical Systems, Pyeongtaek, Korea). Heparin (5 ml; 100 U/kg; JW Pharm Co., Seoul, Korea) was introduced through the arterial sheath. Preprocedural angiography was performed to assess the condition of both iliac arteries. The stent delivery system with a loaded closed-cell SEMS was inserted into the iliac artery, and the stent was placed in at the proximal iliac bifurcation. Postprocedural angiography was performed via the sheath to confirm stent patency and location. Pre- or postprocedural balloon angioplasty was not performed to evaluate the effects of the closed-cell SEMS itself. Percutaneous hemostasis was performed using a vascular closure device (FemoSeal, Vascular Closure System, Terumo Co.) and manual compression by hand for 10 min. All animals received aspirin (100 mg; Bayer AG, Leverkusen, Germany) and clopidogrel (Plavix Tab 75 mg; HANDOK Inc., Seoul, Korea) daily from 3 days before the procedure until the end of the study. Antibiotics (gentamicin, 7 mg/kg; Shin Poong Pharm Ltd., Seoul, Korea) and analgesia (keromin, ketorolac 1 mg/kg; Hana Pharm Ltd., Seoul, Korea) were administered for 3 days after the procedure.

### Follow-up angiography

Follow-up angiographies were performed before and after the procedure, and immediately before sacrifice at 4 weeks. Preprocedural angiography was performed to observe the proximal and distal landing zones for stent placement. Follow-up angiography via the right carotid artery was also performed to evaluate stent patency and late stent-related complications at 4 weeks after the procedure. Luminal diameters were measured and compared at the proximal, middle, and distal portions of the stented iliac arteries using RadiAnt DICOM viewer (version 2020.2; Medixant, Poznan, Poland).

### Gross and histological examinations

Surgical exploration of the abdominal aortic bifurcation and both iliac arteries was performed for gross examination of possible arterial injury and presence of thrombosis following stent placement. Approximately 100 ml of heparin-treated saline (100 U/kg, JW Pharm Co., Seoul, Korea) was irrigated into the extracted arteries. The stented iliac arteries and the normal control contralateral arteries were extracted for histological examination. Stent-grafts were gently removed from stented arteries for apparent surface thrombus. The degree of thrombus formation was determined using a thrombogenicity score of 1 (mild), thrombus nonexistent or minimal; 2 (mild-to-moderate), thrombus minimal, observed to cover 1–25% of the material surface; 3 (moderate), thrombus moderate, observed to cover 26–50% of the material surface; 4 (moderate-to-severe), thrombus severe, observed to cover 51–75% of the material surface; and 5 (severe), thrombus extensive, covering 76–100% of the material surface^19^. Samples were perfused for 48 h with 4% formalin for fixation. Thereafter, samples were axially sectioned in the middle portion of the stented iliac artery. Tissue samples were embedded in paraffin, cut into 5 μm-thick slices, and stained with hematoxylin and eosin (H&E) and Masson’s trichrome (MT). Histological evaluation using H&E staining was performed to determine the thickness of neointimal hyperplasia and the degree of inflammatory cell infiltration. In addition, the degree of collagen deposition was determined in MT-stained sections. Thickness of neointimal hyperplasia and degree of inflammatory cell infiltration were calculated as the average of eight values around the circumference. Degrees of inflammatory cell infiltration and collagen deposition were subjectively classified as 1: mild, 2: mild to moderate, 3: moderate, 4: moderate to severe, and 5: severe according to the distribution of inflammatory cells and collagen^20^. All stained samples were scanned using a digital slide scanner (Pannoramic 250 FLASH III, 3DHISTECH Ltd., Budapest, Hungary). Measurements were obtained using CaseViewer (3DHISTECH, Ltd., Budapest, Hungary). Analyses of histological findings were based on the consensus of three observers who were blinded to the study.

### Statistical analysis

Data were expressed as mean ± standard deviation (SD). Differences between groups were analyzed using two-sample t-test and Mann–Whitney U test, as appropriate. *p* < 0.05 was considered statistically significant. Statistical analyses were performed using SPSS software (version 27; IBM, Chicago, USA).

### Approval for animal experiments

This study was approved by the Institutional Animal Care and Use Committee (IACUC approval number 2022-13-143) and conformed to US National Institute of Health guidelines for humane handling of laboratory animals. The study was carried out in compliance with the ARRIVE guidelines.

## Results

### Mechanical characteristics

The results of the bench-top tests are presented in Fig. 2. The force-diameter in response to the radial force was numerically higher in C-SEMS compared to B-SEMS (37.2 N/mm vs. 32.5 N/mm) (Fig. 2b). Furthermore, the force–displacement in response to crush resistance (4.1 N/mm vs. 3.7 N/mm), cycle (3.9 N/mm vs. 3.5 N/mm), and three-point bending (0.11 N/mm vs. 0.08 N/mm) were numerically higher in C-SEMS than in B-SEMS (Fig. 2c–e).

### Procedural outcomes and angiographic findings

**Figure 3:**
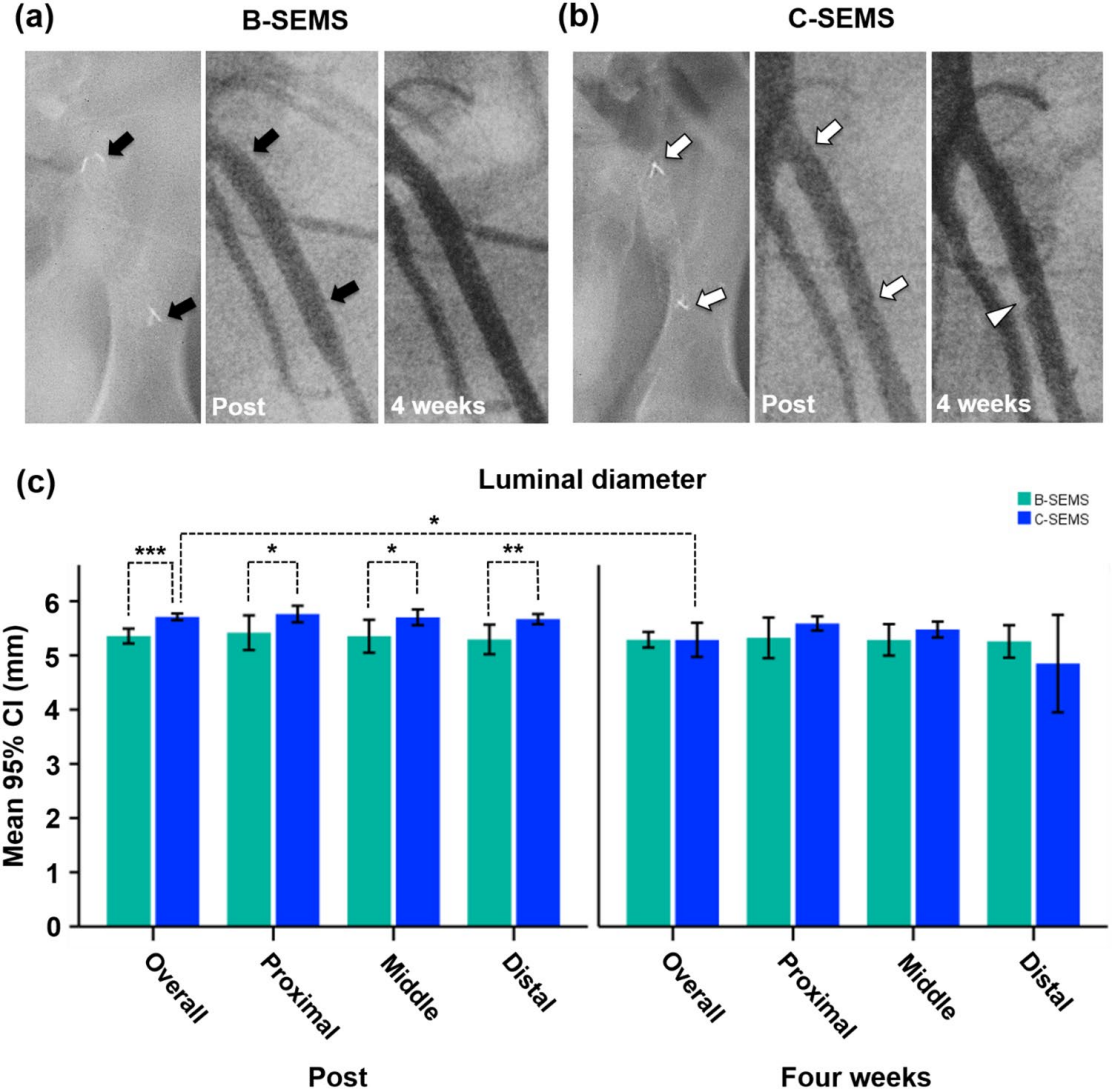
Representative follow-up angiographies of mean luminal diameters in stented iliac arteries. Follow-up angiographies obtained immediately, and 4 weeks after stent placement showing good patency of (**a**) B-SEMS (*black arrows*) and (**b**) C-SEMS (*white arrows*). Follow-up angiography after 4 weeks showed mild luminal narrowing (*white arrowhead*) at the distal portion. (**c**) Graph showing mean luminal diameters in stented iliac arteries. CI, confidence interval; *Note* B-SEMS: bare closed-cell self-expandable metallic stent, C-SEMS: covered closed-cell self-expandable metallic stent; *: *p* < 0.05, **:* p* < 0.01, ***: *p* < 0.001.

All stents were successfully placed in the porcine iliac arteries. The technical success rate was 100% without procedure-related complications. All pigs survived until the end of the study, without further complications. The angiographic findings are shown in Fig. 3. Follow-up angiography showed a good passage of contrast medium through the SEMSs in all pigs without stent-related complications, including endoleak, stent migration, kinking, collapse, and occlusion. Angiographic findings in the B-SEMS group, there was not significantly different between the mean luminal diameter immediately after the procedure and at 4 weeks follow-up. In the C-SEMS group, angiographic findings of the mean overall luminal diameter immediately after the procedure (5.71 ± 0.12 mm) was significantly decreased compared to that at 4 weeks follow-up (5.29 ± 0.63 mm, *p* = 0.012). However, angiography showed no significant differences in the mean luminal diameters in the proximal, middle, and distal portions immediately after the procedure and at 4 weeks follow-up. Significant differences in overall luminal diameters immediately after the procedure were noted between the B-SEMS and C-SEMS groups (5.36 ± 0.27 vs. 5.71 ± 0.12, *p* < 0.001). In addition, luminal diameters were significantly lower in the proximal, middle, and distal portion of the B-SEMS group than in the C-SEMS group (*p* < 0.05). However, angiographic findings of mean luminal diameters at 4 weeks follow-up did not differ significantly between the B-SEMS and C-SEMS groups.

### Gross and histological findings

**Figure 4:**
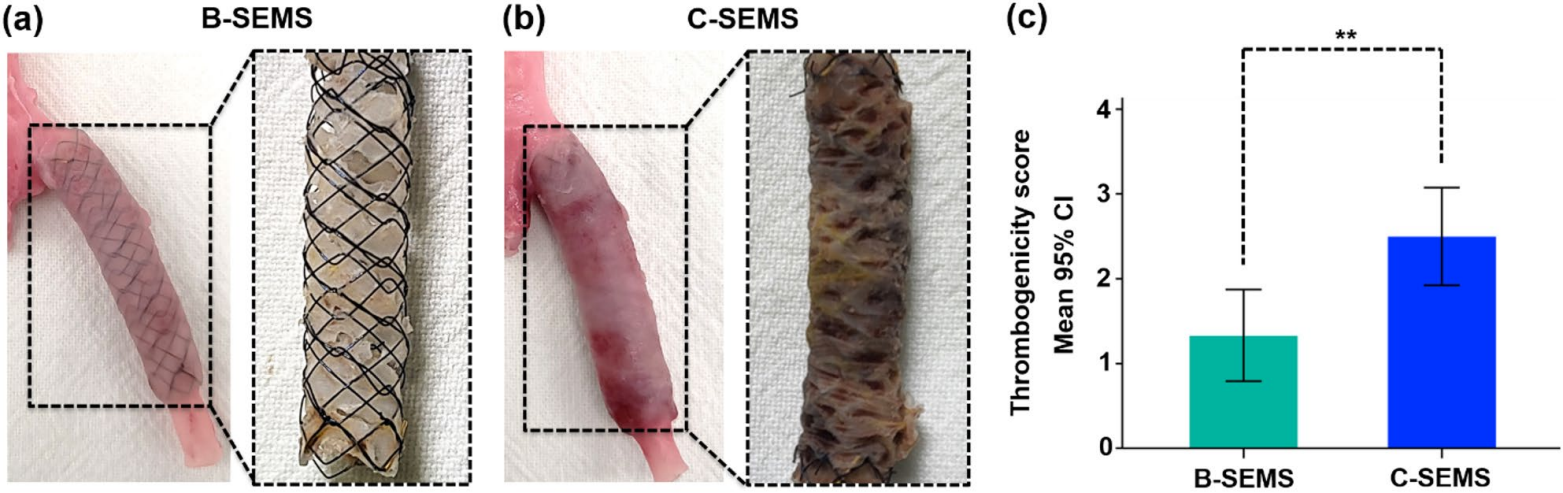
Representative images of gross specimens in the B-SEMS and C-SEMS groups. (**a**) Photographs showing extracted B-SEMS from the artery with glossy white thin neointima tissue. (**b**) Photographs showing extracted C-SEMS with mild thrombus on the inner and outer walls of the C-SEMS. (**c**) Thrombogenicity score in the C-SEMS group was significantly higher than in the B-SEMS group. *Note* CI, confidence interval; *Note* B-SEMS: bare closed-cell self-expandable metallic stent, C-SEMS: covered closed-cell self-expandable metallic stent; **: *p* < 0.01.

All stented iliac arteries were successfully extracted. On gross examination, the inner surface of the B-SEMS group was covered with glossy white thin neointimal tissues. However, thrombus was not observed (Fig. 4a). Thrombus formation was observed on the inner and outer wall of the stents in the C-SEMS group (Fig. 4b). Thrombogenicity score in the C-SEMS group was significantly higher at 4 weeks than that in the B-SEMS group (2.50 ± 0.55 vs. 1.33 ± 0.52, *p* = 0.004) (Fig. 4c).

**Figure 5:**
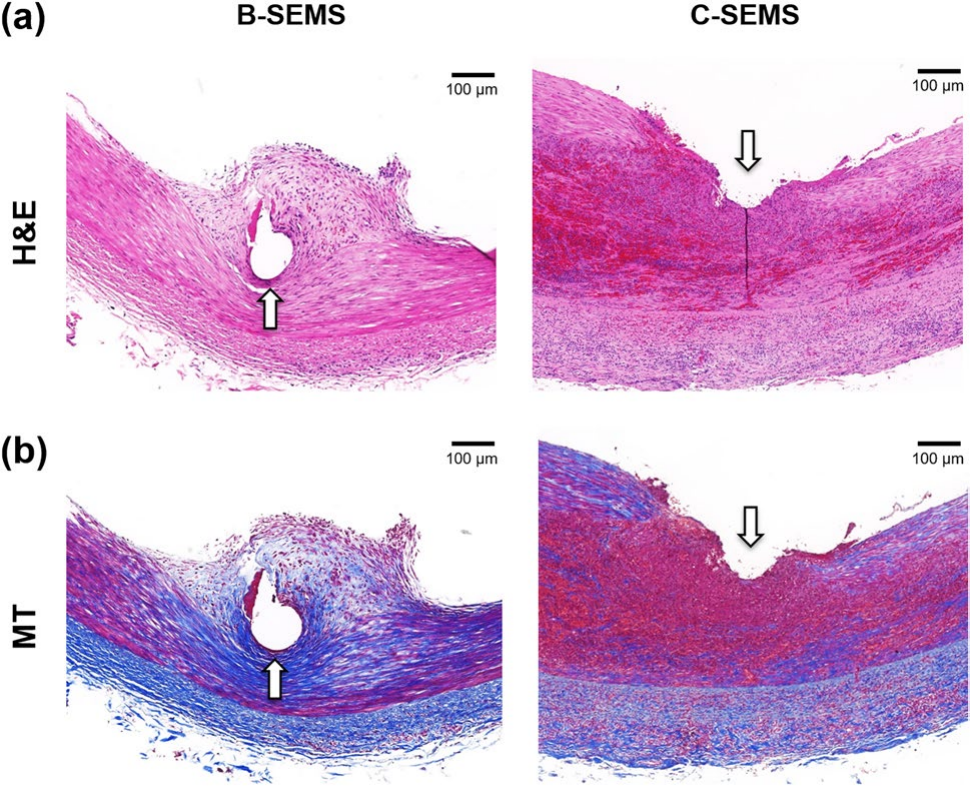
Representative microscopic images of histologic slices obtained with (**a**) hematoxylin and eosin and (**b**) Masson trichrome stains. Degrees of neointimal hyperplasia, inflammatory cell infiltration, and collagen deposition were significantly higher in the C-SEMS group compared to the B-SEMS group. *Note* Arrow: stent struts, B-SEMS: bare closed-cell self-expandable metallic stent, C-SEMS: covered closed-cell self-expandable metallic stent.

**Figure 6:**
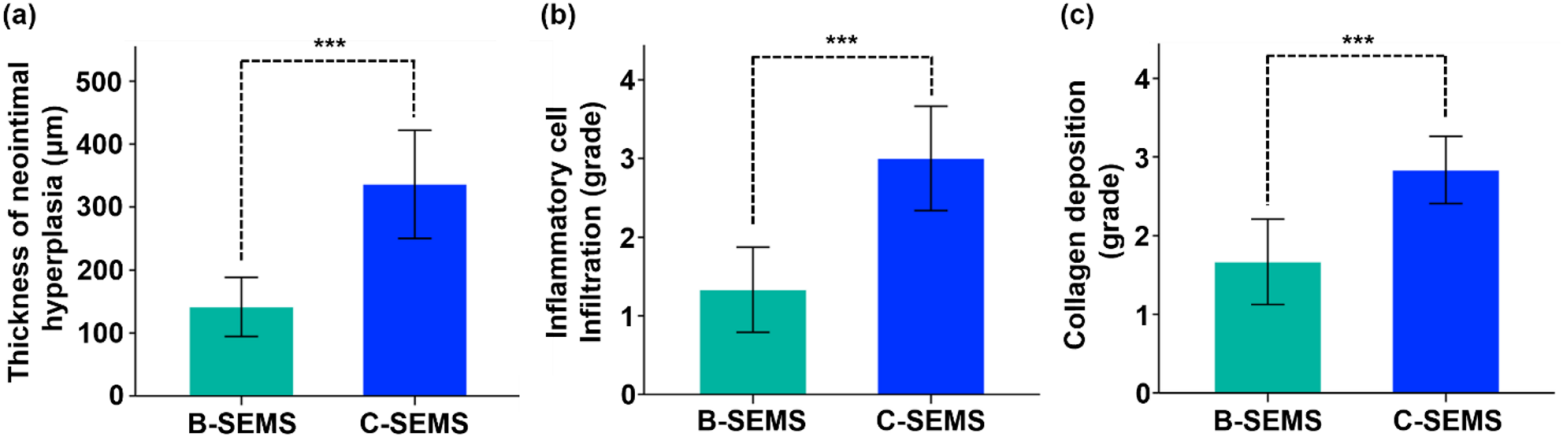
Histological findings. (**a**–**c**) Histological results of stented iliac artery at 4 weeks follow-up. 95% CI, confidence interval; Note. B-SEMS: bare closed-cell self-expandable metallic stent, C-SEMS: covered closed-cell self-expandable metallic stent; ***: *p* < 0.001.

Histological findings are presented in Figs. 5, 6, and Supplementary Fig. 1. The mean (± SD) thickness of neointimal hyperplasia in the C-SEMS group (336.2 ± 81.7 μm) was significantly higher than that in the B-SEMS group (141.6 ± 44.8 μm) (*p* < 0.001) (Fig. 6a). Moreover, the degree of inflammatory cell infiltration (3.00 ± 0.63 vs. 1.33 ± 0.52, *p* < 0.001) (Fig. 6b) and degree of collagen deposition (2.83 ± 0.41 vs. 1.67 ± 0.52, *p* < 0.001) (Fig. 6c) were significantly higher than that in the C-SEMS group at 4 weeks compared to the B-SEMS group.

## Discussion

The results of the present study demonstrated that the patency of closed-cell B-SEMS and C-SEMS was well preserved for 4 weeks without stent-related complications. Although one case showed mild luminal narrowing with a thrombus in the distal portion of the C-SEMS, stent occlusion was not observed in any of the cases. Consistent with the gross findings, histological results demonstrated that neointimal hyperplasia with inflammatory cell infiltration and collagen deposition in the C-SEMS group was significantly higher than that in the B-SEMS group. Slightly greater neointimal tissue formation was observed in the B-SEMS group, while mild thrombogenicity was observed in the C-SEMS group. However, all closed-cell SEMSs with and without a covering membrane showed good patency of the stented iliac arteries. Furthermore, angiographic findings in the B-SEMS group, there was not significantly different between the mean luminal diameter immediately after the procedure and at 4 weeks follow-up. However, the overall luminal diameters in C-SEMS groups at 4 weeks follow-up were significantly different from those immediately after the procedure.

Endovascular therapy is a widely used technique that has been recognized as a common treatment for PAD^21^. Early research on PAD interventions focused on balloon angioplasty and angioplasty with balloon-expandable stent placement for endovascular management of arterial stenosis^22^. Recently, self-expandable stents using nitinol material have been used in PAD more often than balloon-expandable stents due to concerns of severe angulation and tortuosity leading to iliac artery injury, including extravasation, dissection, or rupture, following high-pressure balloon inflation^23^. Closed-cell SEMSs are known to be more rigid and, therefore, are more preferably used in straight morphologies. In contrast, the flexible open-cell SEMSs are ideal for tortuous lesions^9^. Moreover, open-cell SEMSs have become standard practice over closed-cell SEMSs, mainly attributing to easier and more precise placement, non-shortening of the open-cell structure, and more prolonged vascular patency^24^. However, Alparslan et al. reported no significant differences in periprocedural complications between the open- and closed-cell SEMSs in 155 patients undergoing carotid artery stenting. Although in-stent restenosis was more common in the open-cell group, clinical outcomes turned out to be comparable^25^.

Mechanical properties are critical to optimize stent performance for endovascular therapy, and an ideal stent needs to resist high mechanical loading in the vessels^26^. In this study, the resultant radial force, crush resistance, cycle, and bending behaviors of the investigated SEMSs confirmed the usefulness of the closed-cell design and the hand-knitted technique. Although the overall mechanical properties of B- and C-SEMSs were similar, the radial, crush resistance, cycle, and three-point bending forces were slightly higher in C-SEMS than in B-SEMS. Dabir et al.^27^ reported that in terms of radial force, the closed-cell SEMS containing a smaller free-cell area between the stent strut resulted in a higher rigidity compared with the open-cell SEMS. Laasch et al.^28^ demonstrated lower radial force (5.6 N/mm) when the closed-cell SEMS was woven compared with the hand-knitted one (33.4 N/mm). The radial force of the stent in combination with the chosen stent-to-vessel diameter ratio influence the interaction between the stent and arterial wall, potentially leading to arterial wall injury^29^. Based on our bench-top experimental findings, that the hand-knitted closed-cell SEMS exhibited superior resistance to high stress, maintained its self-expanding characteristics, and possessed adequate strength to ensure arterial lumen patency.

The covered stent, originally intended for aneurysms or arterial ruptures, is now commonly used for aortoiliac occlusive disease, as it offers a potential advantage in preventing in-stent restenosis and reducing the risk of distal embolization in complex disease^30–32^. Covered stents with e-PTFE membranes prevent the exposure of macrophages to atherosclerotic tissue, reduce cytokine and growth factor secretion, and directly block smooth muscle cell migration and neointimal tissue growth^33,34^. Additionally, they have been associated with improved flow patterns and a lower risk of thrombus formation compared to bare metal stents^35^. Owing to these various options, it is becoming increasingly important to select the appropriate stent type to improve stent patency and hemocompatibility, and reduce complications. However, angiographic findings of the C-SEMS group revealed mild luminal narrowing with thrombosis in the distal portion of the stent and the overall luminal diameter decreased at 4-weeks follow-up compared with that immediately after the procedure. Conventional covered stents by e-PTFE or polyethylene terephthalate have been used for the endovascular treatment^36^. However, in-stent restenosis frequently occurred in the arteries of relatively small diameters (< 6 mm) caused by increased risk of calcification or thromboembolism when the covering membrane was present^34^. Wong et al.^37^ reported that at 4-weeks after placing an open-cell SEMS covered with e-PTFE, the luminal diameter of the porcine carotid artery decreased by 67.2% due to neointimal hyperplasia with inflammation and thrombosis. The risk of thromboembolism might be increased when placing the C-SEMS covered with e-PTFE in the small-diameter arteries.

Recently, closed-cell SEMSs using nitinol wire have been hand-knitted into a tubular diamond-cell configuration. The results of this study showed that all closed-cell SEMS, with or without an e-PTFE covering membrane, were successfully placed into the porcine iliac artery. Procedural and angiographic findings showed good short-term patency without procedure- and stent-related complications such as endoleak, stent migration, kinking, collapse, and/or occlusion. Closed-cell SEMSs cover a greater percentage of the vascular wall in the stented region and thereby have less exposed cell area, which offers greater scaffolding potential^4,25^. Our preliminary study revealed that closed-cell SEMSs was successfully maintained the patency without significant stent-in-restenosis for 4 weeks.

This study has several limitations. First, the follow-up period might be too short to meaningful evaluate neointimal proliferation and thrombus formation. Second, a heterogeneous difference between the actual diameter of iliac arteries and the size of closed-cell SEMSs was observed. This size difference may induce different degrees of neointimal hyperplasia owing to stretching by an oversized closed-cell SEMS. Third, a normal iliac artery model was used. These findings may not reflect all pathological mechanisms that occur in humans following stent placement; however, the porcine model is considered an anatomically suitable model for preclinical evaluation of stent placement. Fourth, biocompatibility of the closed-cell SEMS used in this study was not evaluated. Finally, the comparison between the closed-cell and open-cell or commercially available SEMSs was not made previous studies were referred to instead. Although further preclinical studies were required to validate the long-term outcomes of the closed-cell SEMS, our study supports the premise that the hand-knitted closed-cell SEMS can successfully maintain stent patency during follow-up.

The closed-cell SEMSs successfully maintained patency for 4 weeks without stent-related complications in the porcine iliac artery. Although a mild thrombus with neointimal hyperplasia was observed in the C-SEMS group, subsequent occlusion, and in-stent stenosis did not occur in any of the pigs until the end of the study. Closed-cell SEMS, with or without the e-PTFE covering membrane, was effective and safe for the porcine iliac artery. Although additional studies are required to further validate the current data, these findings may provide sufficient evidence for the use of closed-cell SEMSs in the iliac artery to treat PAD.

## Supplementary Information

Supplementary Figures.

## Data Availability

The datasets generated and/or analyzed during the current study are available from the corresponding author upon reasonable request.

## References

[1] Gerhard-Herman MD (2017). AHA/ACC guideline on the management of patients with lower extremity peripheral artery disease: Executive summary: A report of the American College of Cardiology/American Heart Association task force on clinical practice guidelines. Circulation.

[2] Mozaffarian D, Writing Group Members (2016). Heart disease and stroke statistics-2016 update: A report from the American Heart Association. Circulation.

[3] Jongkind V, Akkersdijk GJ, Yeung KK, Wisselink W (2010). A systematic review of endovascular treatment of extensive aortoiliac occlusive disease. J. Vasc. Surg..

[4] Sommer CM (2010). Impact of stent design on in-stent stenosis in a rabbit iliac artery model. CardioVasc. Interv. Radiol..

[5] AbuRahma AF, Hayes JD, Flaherty SK, Peery W (2007). Primary iliac stenting versus transluminal angioplasty with selective stenting. J. Vasc. Surg..

[6] Scheinert D, Scheinert S, Sax J, Piorkowski C, Braunlich S, Ulrich M (2005). Prevalence and clinical impact of stent fractures after femoropopliteal stenting. J. Am. Coll. Cardiol..

[7] Lammer J (2015). Sustained benefit at 2 years for covered stents versus bare-metal stents in long SFA lesions: The VIASTAR trial. Cardiovasc. Interv. Radiol..

[8] Humphries MD, Armstrong E, Laird J, Paz J, Pevec W (2014). Outcomes of covered versus bare-metal balloon-expandable stents for aortoiliac occlusive disease. J. Vasc. Surg..

[9] Maintz D (2006). 64-slice multidetector coronary CT angiography: In vitro evaluation of 68 different stents. Eur. Radiol..

[10] Stoeckel D, Bonsignore C, Duda S (2002). A survey of stent designs. Minim. Invas. Ther. Allied Technol..

[11] Kang CH (2016). Comparison of open-cell stent and closed-cell stent for treatment of central vein stenosis or occlusion in hemodialysis patients. Iran. J. Radiol..

[12] Lakhter V, Aggarwal V (2017). Current status and outcomes of iliac artery endovascular intervention. Interv. Cardiol. Clin..

[13] Mwipatayi BP (2011). A comparison of covered vs bare expandable stents for the treatment of aortoiliac occlusive disease. J. Vasc. Surg..

[14] Ryu DS (2022). Efficacy of thermoplastic polyurethane and gelatin blended nanofibers covered stent graft in the porcine iliac artery. Sci. Rep..

[15] Matsumoto T (2016). Radial force measurement of endovascular stents: Influence of stent design and diameter. Vascular.

[16] International Organization for Standardization. ISO 25539–2:2012: Cardiovascular implants: Endovascular devices: Part 2: Vascular stents (2012).

[17] International Organization for Standardization. ISO 7198:2016: Cardiovascular implants and extracorporeal systems—Vascular prostheses—Tubular vascular grafts and vascular patches.

[18] International Organization for Standardization. ISO 25539-1:2017: Cardiovascular implants: Endovascular devices: Part 1: Endovascular prostheses (2017).

[19] Wolf MF (2021). In vitro methodology for medical device material thrombogenicity assessments: A use condition and bioanalytical proof-of-concept approach. J. Biomed. Mater. Res. B Appl. Biomater. Res..

[20] Park JH (2019). Histologic analysis with the newly designed exoskeleton Seal(®) stent-graft in the porcine abdominal aorta. Cardiovasc. Intervent. Radiol..

[21] Aboyans V (2018). ESC guidelines on the diagnosis and treatment of peripheral arterial diseases, in collaboration with the European Society for Vascular Surgery (ESVS): Document covering atherosclerotic disease of extracranial carotid and vertebral, mesenteric, renal, upper and lower extremity arteries endorsed by: The European Stroke Organization (ESO) The task force for the diagnosis and treatment of peripheral arterial diseases of the European Society of Cardiology (ESC) and of the European Society for Vascular Surgery (ESVS). Eur. Heart J..

[22] Violari E (2022). Endovascular treatment of infrainguinal peripheral arterial disease (pad): Update on stent technology. Tech. Vasc. Interv. Radiol..

[23] Choi WG (2016). Study design and rationale of the 'Balloon-Expandable Cobalt Chromium SCUBA Stent versus Self-Expandable COMPLETE-SE Nitinol Stent for the Atherosclerotic ILIAC Arterial Disease (SENS-ILIAC Trial) Trial': Study protocol for a randomized controlled trial. Trials.

[24] Bismuth J (2017). Pivotal study of a next-generation balloon-expandable stent-graft for treatment of iliac occlusive disease. J. Endovasc. Ther..

[25] Stockx L (2010). Express LD vascular stent in the treatment of iliac artery lesions: 24-month results from the MELODIE trial. J. Endovasc. Ther..

[26] Ansari F, Pack LK, Brooks SS, Morrison TM (2013). Design considerations for studies of the biomechanical environment of the femoropopliteal arteries. J. Vasc. Surg..

[27] Darius D (2018). Physical properties of venous stents: An experimental comparison. Cardiovasc. Intervent. Radiol..

[28] Laasch HU (2020). ‘Radial force’ of colonic stents: A parameter without consistency, definition or standard. Int. J. Gastrointest. Interv..

[29] Maleckis K (2018). Stents in the femoropopliteal artery: A mechanical perspective on material, design, and performance. Ann. Biomed. Eng..

[30] Bekken JA, Jongsma H, de Vries JP, Fioole B (2014). Self-expanding stents and aortoiliac occlusive disease: A review of the literature. Med Devices..

[31] Dolmatch B, Dong YH, Heeter Z (2007). Evaluation of three polytetrafluoroethylene stent-grafts in a model of neointimal hyperplasia. J. Vasc. Interv. Radiol..

[32] Grimme FA, Goverde PA, Van Oostayen JA, Zeebregts CJ, Reijnen MM (2012). Covered stents for aortoiliac reconstruction of chronic occlusive lesions. J. Cardiovasc Surg..

[33] Groot Jebbink E (2015). Geometrical consequences of kissing stents and the covered endovascular reconstruction of the aortic bifurcation configuration in an in vitro model for endovascular reconstruction of aortic bifurcation. J. Vasc. Surg..

[34] Isenberg BC, Williams C, Tranquillo RT (2005). Small-diameter artificial arteries engineered in vitro. Circ. Res..

[35] Wong G (2008). Inhibition of experimental neointimal hyperplasia by recombinant human thrombomodulin coated ePTFE stent grafts. J. Vasc. Surg..

[36] Tatli E (2017). Comparison of closed-cell and hybrid-cell stent designs in carotid artery stenting: Clinical and procedural outcomes. Postepy Kardiol Interwencyjnej..

[37] Alparslan B (2016). The effect of stent cell geometry on carotid stenting outcomes. Cardiovasc. Intervent. Radiol..

