# Supplementary material for: Efficacy of closed cell self expandable metallic stent for peripheral arterial disease in the porcine iliac artery
Source: Sci Rep. 2023 May 26;13:8601. doi: 10.1038/s41598-023-35878-y (PMC10219949; doi:10.1038/s41598-023-35878-y)
Supplement: Supplementary file 1 — Supplementary Figures. [file 41598_2023_35878_MOESM1_ESM.pdf]

Supplementary Information

Efficacy of Closed cell Self expandable Metallic Stent for Peripheral Arterial Disease in the Porcine Iliac Artery

Authors: Dae Sung Ryu[1†], Dong-Sung Won[1†], Ji Won Kim[1], Yubeen Park[1], Song Hee Kim[1], Jeon Min Kang[1], Chu Hui Zeng[1], Dohyung Lim[2], Hyun Choi[2*], Jung-Hoon Park[1*]

Affiliations:

[1]Biomedical Engineering Research Center, Asan Institute for Life Sciences, Asan Medical Center, 88 Olympic-ro 43-gil, Songpa-gu, Seoul, 05505, Republic of Korea

[2]Department of Mechanical Engineering, Sejong University, 209, Neungdong-ro, Gwangjin-gu, Seoul, 05006, Republic of Korea

[†]D.S.R., and D.-S.W. contributed equally to this work and are co-first authors.

[*]H.C., and J.-H.P. contributed equally to this work and are co-corresponding authors.

Correspondence: Jung-Hoon Park, Ph.D.[1], Hyun Choi[2]

[1]Biomedical Engineering Research Center, Asan Institute for Life Sciences, Asan Medical Center, 88 Olympic-ro 43-gil, Songpa-gu, Seoul, 05505, Republic of Korea

Tel: +82-2-3010-4123

Fax: +82-2-476-0090

E-mail: jhparkz@amc.seoul.kr

[2]Department of Mechanical Engineering, Sejong University, 209, Neungdong-ro, Gwangjin-gu, Seoul, 05006, Republic of Korea

Tel: +82-2-3408-3672

Fax: +82-2-3408-4333

E-mail: godsknight96@hanmail.net

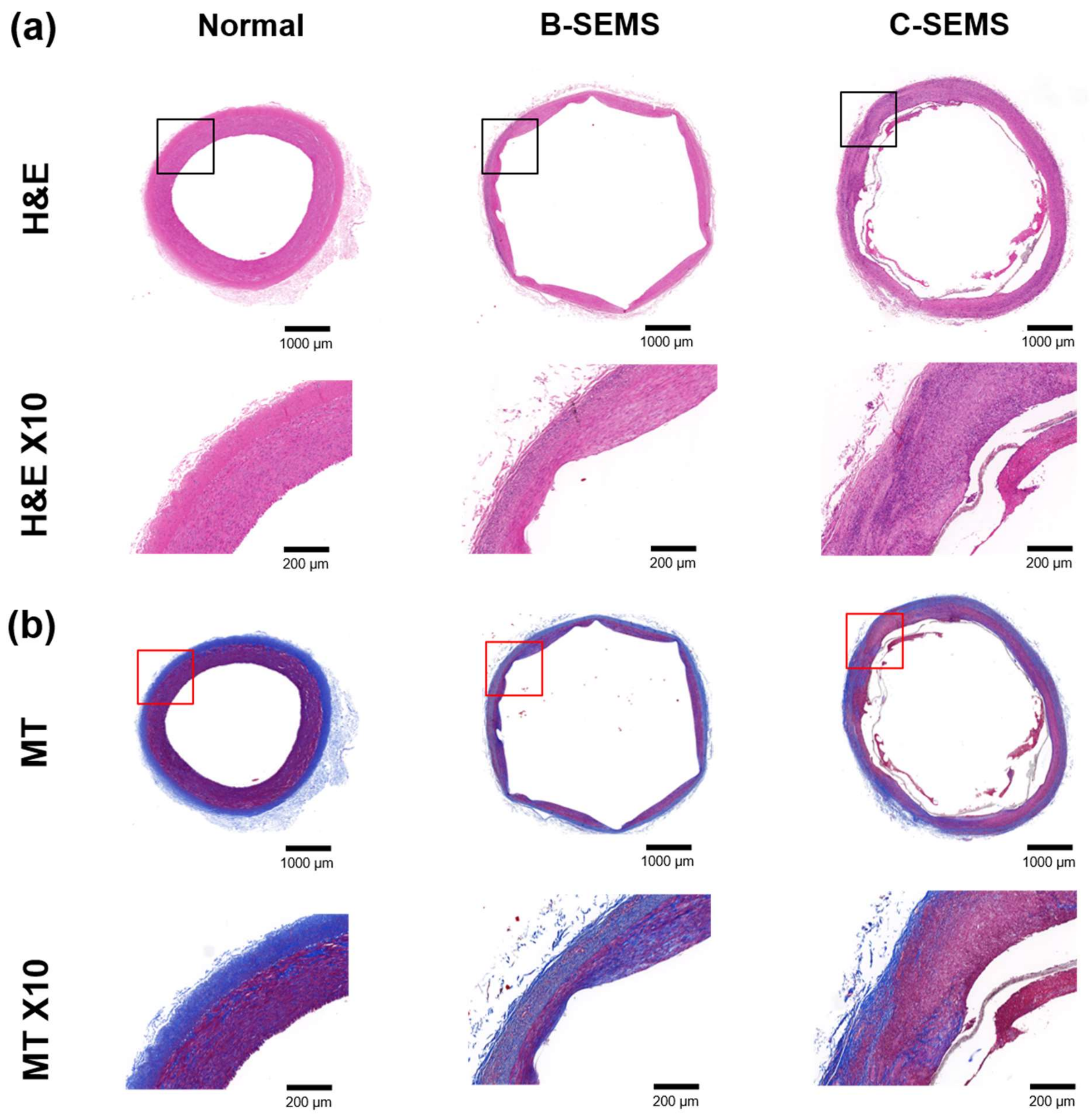

Supplementary Figure 1. Representative images showing serial histological changes in normal iliac artery, B-SEMS, and C-SEMS. (a) H&E staining with X 2 and X 10 magnifications. X 10 images indicate locations for the higher magnification (*black boxes*). (b) MT staining with X 2 and X 10 magnifications. X 10 images indicate locations for the higher magnification (*red boxes*). Note. B-SEMS: bare closed-cell self-expandable metallic stent, C-SEMS: covered closed-cell self-expandable metallic stent, H&E: hematoxylin and eosin, MT: Masson trichrome
